# The Association of Human Milk Proportion with the Clinical Outcomes of Necrotizing Enterocolitis in Preterm Infants: A Retrospective Study

**DOI:** 10.3390/nu15173796

**Published:** 2023-08-30

**Authors:** Keqin Liu, Jinjin Guo, Jixin Yang, Yanwei Su

**Affiliations:** 1 School of Nursing, Tongji Medical College, Huazhong University of Science and Technology, Wuhan 430030, China; liukeqin@hust.edu.cn (K.L.); guojinjin@hust.edu.cn (J.G.); 2 Department of Pediatric Surgery, Tongji Hospital, Tongji Medical College, Huazhong University of Science and Technology, Wuhan 430030, China; yangjixin0910@126.com

**Keywords:** human milk, preterm infants, necrotizing enterocolitis, health outcome, quantitative methods

## Abstract

Human milk (HM) has been associated with a lower risk of necrotizing enterocolitis (NEC). However, the association of precise HM proportion with the outcome of NEC remains unclear. A total of 77 cases and 154 matched controls were included in this study. The samples were divided into three groups based on the HM proportion of the total enteral intake before NEC onset: ≥70% (HHM), <70% (LHM), and 0% (NHM). The study cohort did not show a significant association between different HM proportions and NEC risk. The adjusted odds ratio (OR) for the highest versus the lowest intake was 0.599. In the prognosis of NEC, different HM proportions significantly affected weight gain, the timing of NEC onset, diagnosis time, hospitalization cost, and the severity of NEC (*p* < 0.05). Our findings support the beneficial effects of HM on reducing NEC in preterm infants, particularly when a greater proportion of HM of the total enteral intake is included in their feeding. Additionally, the study indicates that preterm infants fed with lower proportions of HM of the total enteral feeding are more prone to experiencing severe cases of NEC.

## 1. Introduction

Necrotizing enterocolitis (NEC) is one of the most devastating diagnoses seen primarily in premature infants, which is associated with high morbidity and mortality [1]. NEC has an ambiguous onset and a rapid progression, making it difficult to detect in the early stages. It typically begins with symptoms like feeding intolerance or abdominal distention, which gradually escalate into severe complications such as neonatal sepsis, disseminated intravascular coagulation, and death. Studies have shown that the majority of NEC (>90%) occurs in preterm infants, and it has a mortality rate as high as 20–45% [2,3]. Therefore, it is important to identify preterm infants with high-risk factors for NEC to prevent this disease precociously. Historically, the high incidence of NEC among preterm infants has been mainly attributed to prematurity and low birth weight [4,5]. As a multifactor disease, intestinal immaturity, intestinal barrier disruption, intestinal immune immaturity, and inflammatory cascades have been confirmed to contribute to the development of NEC [6].

There is growing evidence demonstrating that human milk (HM) has a protective effect against NEC [7,8,9]. For instance, human milk growth factors could regulate gut barrier integrity and microbial colonization, and human milk oligosaccharides (HMOs) could attenuate inflammatory responses and provide immunological defenses [10,11]. Studies found that HM is rich in bifidobacterial and Bacteroides, which can protect the intestinal mucosa and reduce the risk of feeding intolerance [12]. Studies have shown that the feeding of HM to preterm infants is differentially associated with the onset and development of NEC. Miller et al. stated that any HM compared to formula resulted in a 4% reduction in NEC and that a greater HM percent in the diet had a protective effect against NEC [13]. Observational studies also reported that HM reduced sepsis and NEC, thus improving outcomes of preterm infants [8]. These findings demonstrate that HM should be recommended as the optimal feeding for preterm infants.

Previous studies have also found that intestinal development in premature infants plays a major role in its pathogenesis. Preterm infants are susceptible to gastrointestinal mucosal damage and feeding intolerance due to their immunocompromised state. The intestinal mechanical barrier is mainly achieved by tight junctions (TJs) and adherence junctions (AJs) of the gastrointestinal epithelium, which prevent intestinal mucosa from luminal noxious molecules entering through blood circulation. However, preterm infants have fewer AJs and are vulnerable to environmental influences, thus leading to disruption of the intestinal barrier and aggravating gut damage [14]. The intestinal immunity of premature infants can be divided into innate and adaptive immunities. The imbalance of the maturation of intestinal innate and adaptive immune defense mechanisms contributes to the incidence and development of NEC, which is mainly through Toll-like receptors (TLRs) [15]. Recent evidence has suggested that TLR-2 attenuates inflammatory damage to the intestinal mucosa via *Lactobacillus*, while when TLR-4 is negatively regulated, the activation of nuclear transcription factors and the inflammatory injury of intestinal mucosa are reduced [16,17]. TLRs can stimulate the inflammatory response, induce intestinal epithelial cell apoptosis, and inhibit intestinal mucosal repair, thereby aggravating intestinal injury in preterm infants with NEC.

Despite the many benefits of HM, most preterm infants are fed with a mixture of HM and formula. The global rate of exclusive HM is low at 33%. Exclusive HM rates at 6 months differ across the globe, ranging from 1% in the UK to 21% in China [18]. This could be attributed to the low willingness of mothers of preterm infants to breastfeed, insufficient HM production, and a lack of donated human milk [19]. To date, studies on the benefits of HM in premature infants have mainly compared a mother’s own milk with premature infant formula, confirming a significant protective effect of HM against NEC [20,21,22]. However, few studies have evaluated the effects of precise HM proportion on the development and outcome of NEC in preterm infants [23]. Whether partial HM reduces the occurrence and severity of NEC in preterm infants remains uncertain.

To analyze the effects of different HM proportions of the total enteral feeding on NEC in preterm infants, a retrospective study was conducted. We hypothesized that feeding variables would be associated with the occurrence of NEC, and the protective effect of a high HM proportion would be most pronounced in preterm infants. We aimed to (i) analyze the associations of different HM proportions of the total enteral intake with the occurrence of NEC in preterm infants and to (ii) evaluate the clinical outcome and disease severity in NEC infants receiving different HM volumes of the total enteral feeding (no, low, and high HM proportions).

## 2. Methods

### 2.1. Study Population

**Figure 1:**
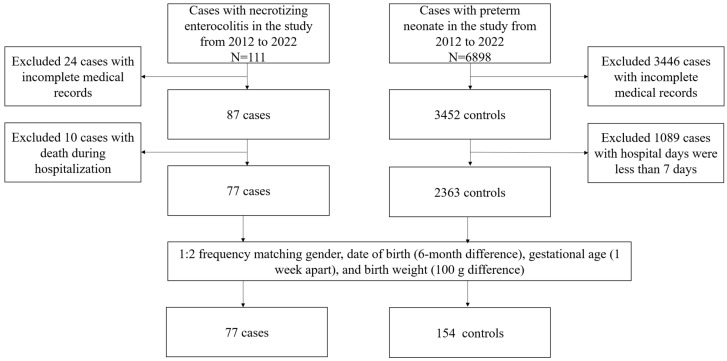
Flow chart of selection of the study population.

We conducted a retrospective study in a neonatal intensive care unit (NICU). The study population consisted of all preterm infants hospitalized in the NICU from a single institution between January 2012 and January 2022. Preterm was defined as neonates born alive before 37 weeks of pregnancy were completed. The diagnosis of NEC was based on clinical and radiological findings (Bell’s stage I) [24]. Deaths were excluded in this study because NEC itself is a fast-onset disease with rapid disease progression, and we were unable to obtain relevant feeding information in less than 7 days. Two control patients were selected from the database to match with gender, date of birth (6-month difference), gestational age (1 week apart), and birth weight (±100 g difference). A total of 77 cases and 154 matched controls were included in this study (see Figure 1). The present report follows the recommendations of the statement “strengthening the reporting of observation studies in epidemiology” (STROBE).

### 2.2. Data Collection

Data were collected retrospectively using electronic medical records (EMRs). For each eligible sample, the data collection process took place in two steps. First, two researchers independently collected and collated extracted data. Secondly, a second researcher validated all extracted data to ensure accuracy. Completed demographic and clinical data, including gender, gestational age (GA), birth weight, method, Apgar score, related complications, feeding methods, and HM volumes, as well as clinical characteristics, severity, and prognosis associated with NEC, were extracted from the EMRs. Patients were classified as NEC cases if they were identified as having NEC, and their medical records were subsequently reviewed to confirm the presence of clinical or radiographic signs of NEC. These signs included symptoms such as bloody stool, abdominal distension, thrombocytopenia, pneumatosis intestinalis, portal venous air, and pneumoperitoneum. In the NEC cases, clinical characteristic measures were selected as the health outcome with the highest inflammatory measures within 24 h of NEC onset. The severity of the NEC was graded according to Bell’s stage. The prognosis of NEC included improvement or cure of the disease, as well as the treatment being unhealed or abandoned. Maternal and neonatal records were obtained and analyzed, focusing on their feeding factors and the outcomes of NEC.

### 2.3. Association between HM Proportion and NEC

HM intake data were collected from nursing record sheets by recording separately the daily intake of maternal and formula milk. Infants only received HM from their mothers, including fresh mother’s own milk (MOM) and frozen HM (−18 ± 2 °C). All mothers were encouraged to give their milk to their babies. Donor milk was not available in our NICU. Formula milk was given if there was insufficient HM to meet the needs of the infants. From one to two weeks, the milk intake was routinely increased at a speed of 1–2 mL/kg/meal to advance the feed volume slowly. During this time, neonates received most of their fluids and nutrition parenterally, usually in the form of commercially available solutions containing energy, glucose, protein, vitamins, and fat. When HM volume reached 50–100 mL/kg/d, bovine-derived HM fortifier (HMF) was added to the HM until hospital discharge. The proportion of HM was calculated as the total amount of HM intake divided by the total volume of enteral feeding within hospitalization.

Cases and matched controls were defined as presenting or not presenting with NEC. First, all preterm infants included in this study were divided by different HM proportions. Meanwhile, infants diagnosed with NEC were grouped according to different HM proportions. NEC infants were separated into groups based on the proportion of HM intake to total enteral feedings during hospitalization (0%, <70%, ≥70%). Cases receiving more than or equal to a 70% HM proportion were included in the HHM group, those receiving less than 70% were included in the low HM (LHM) group, and those without HM or with pure formula feeding were included in the no HM (NHM) group. Two clinicians with experience in pediatric-related diseases simultaneously confirmed and rediagnosed NEC using Bell stages to grade its severity. The primary outcome was the associations of different HM proportions of the total enteral intake with the occurrence, development, and prognosis of NEC in preterm infants.

### 2.4. Statistical Analysis

Statistical analysis was performed with SPSS software (version 26; Chicago, IL, USA). Baseline characteristics were described using proportions (categorical variables) and mean ± standard error (ordinal and continuous variables). Conditional logistic regression analyses were performed to assess the association between HM proportion and the occurrence of NEC by calculating adjusted matched odds ratios (ORs) and their 95% confidence intervals (CIs). χ^2^, Welch’s *t*, Fisher’s exact, and one-way ANOVA tests were applied to perform univariate analyses of categorical, numerical, and ordinal outcomes, respectively. *p* values less than 0.05 were considered statistically significant.

## 3. Results

### 3.1. General Characteristic

**Figure 2:**
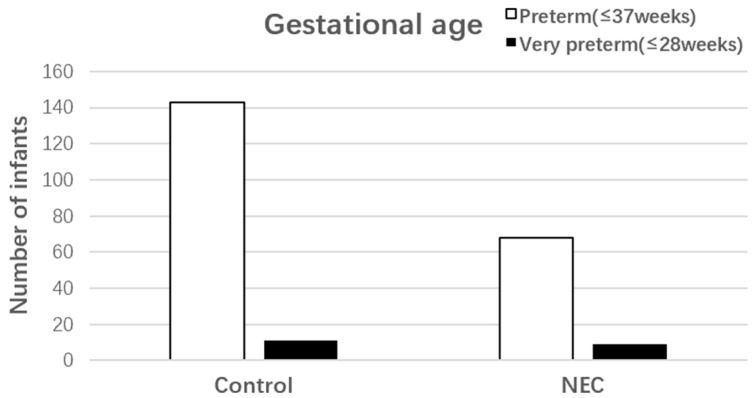
Gestational age of NEC cases and controls.

**Figure 3:**
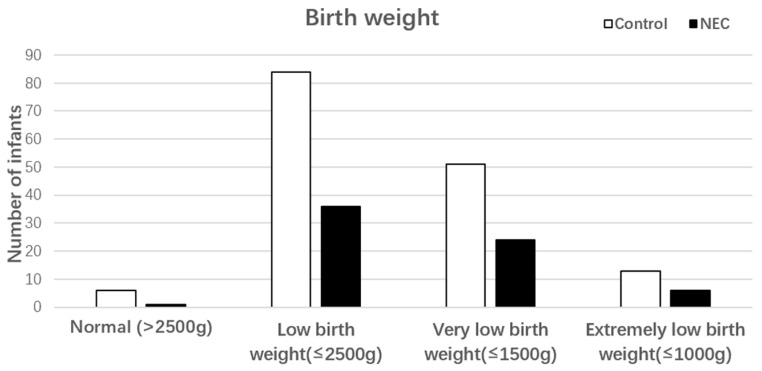
Birth weight of NEC cases and controls.

A total of 77 neonates were identified with NEC, and 154 controls were selected after controlling for data of birth, neonatal sex, gestational age, and birth weight. Of these, 47 (61.04%) were male and 30 (38.96%) were female in the NEC cohort, while 94 (61.04%) were male and 60 (38.96%) were female in the control group. In the NEC cohort, nine (11.68%) were born at ≤28 weeks of gestation, and six (7.79%) had a birth weight of ≤1000 g. In the control group, 11 (7.14%) were born at ≤28 weeks of gestation, and 13 (8.44%) had a birth weight of ≤1000 g (Figure 2 and Figure 3).

The GAs were 31.68 ± 2.60 and 31.77 ± 2.55 weeks, and birth weights were 1660.65 ± 481.36 and 1654.74 ± 483.02 g for the cases and control group, respectively. Demographic information of the NEC cases and control neonates is presented in Table 1. The case–control pairs were comparable for most maternal and neonatal characteristics (Table 1). Maternal records for all NEC cases and controls were reviewed as well. The proportion of preterm infants exposed to neonatal asphyxia (23.38% for cases and 37.66% for controls; *p* = 0.029) was lower in cases who developed NEC compared to controls. Also, the Apgar scores at 1 min for the NEC cohort were 6.90 ± 1.22, with 6.32 ± 1.66 (score) for the control group, and Apgar scores at 5 min were 7.95 ± 1.11 and 7.60 ± 1.22 (score) for the cases and the control group, respectively (*p* < 0.05). Simultaneously, the results show that there were no statistical differences between the two groups for several factors, such as natural birth delivery, gestational diabetes mellitus, etc. (*p* > 0.05).

### 3.2. The Associations of Different HM Proportions and the Occurrence of NEC in Preterm Infants

Table 2 presents the overall crude and adjusted ORs and 95% CIs for NEC according to different HM proportions. Overall, HHM was protective against the risk of the occurrence of NEC (OR < 1). A negative trend was observed between NEC risk and HM proportion in the unadjusted analysis when comparing to the NHM group with an OR = 1.257 (95% CI: 0.565–2.799) for the LHM group with an OR = 0.599 (95% CI: 0.261–1.377) and the HHM group (*p* = 0.317). In the adjusted model controlling for potential confounding factors, the negative association between NEC risk and lower HM proportions was still observed (adjusted OR = 1.045 (95% CI: 0.452–2.417) ) for the LHM group and adjusted OR = 0.573 (95% CI: 0.246–1.336) for the HHM group (*p* = 0.386)).

### 3.3. The Associations of Different HM Proportions with the Outcomes of NEC in Preterm Infants

As shown in Table 3, the clinical characteristics, development, and prognosis of NEC were compared and analyzed. Out of the 77 NEC cases, 11 (≥70% HM intake) were in the HHM group, 13 (<70% HM intake) were included in the LHM group, and 53 (0% HM intake) were in the NHM group.

For weight velocity, the method of Patel et al. [25] was used to calculate g/kg/day, and the formula for that was weight gain (g/kg/d) = [1000 × In (Wt2/Wt1)]/T. Wt2 is discharge weight, Wt1 is birth weight, and T denotes the number of elapsed days between the day of hospital discharge and day of recovery to birth weight. Weight gain is the growth rate in weight during hospitalization. The weight gains in the HHM, LHM, and NHM groups were 14.99 ± 9.41 vs. 9.35 ± 4.14 vs. 8.99 ± 6.88 g/kg/day, respectively. Our results showed significant associations between different HM proportions and weight gain during hospitalization (*p* < 0.05). The onset day was defined as the day when the infant developed any symptoms of abdominal distension, vomiting, bloody stool, or diarrhea. The diagnosis day was when one of the above symptoms was present and the presence of pneumoperitoneum in the intestinal wall or a portal vein or perforation was clear on abdominal plain film or ultrasound. The onset days in the HHM, LHM, and NHM groups were 21.9 ± 12.9 vs.17.9 ± 11.2 vs. 11.7 ± 7.3 days, respectively. The diagnosis days were 25.9 ± 11.7 vs. 19.8 ± 11.3 vs. 13.9 ± 7.9 days in the HHM, LHM, and NHM groups, respectively. These results conclude that onset day and diagnosis day were significantly associated with different HM proportions (*p* < 0.05). Meanwhile, the LHM group had the highest hospitalization cost at CNY 95,554.65 ± 47,002.48. The hospitalization cost of the HHM group was CNY 77,802.46 ± 29,454.21 vs. CNY 64,773.89 ± 49,409.47 for the NHM group. These results indicate that there was a significant association between the different volumes of HM intake and hospitalization costs (*p* < 0.05). In our study, NEC I and NEC II A were classified as “mild” stages, while NEC II B and NEC III were “severe” stages. In the severe group, there were 2 preterm infants (18.18%), 4 preterm infants (30.77%), and 32 preterm infants (60.38%) in the HHM, LHM, and NHM groups, respectively. This finding showed a significant difference in the severity of NEC at various HM volumes (*p* < 0.05).

No statistical difference was found among these three groups in the highest levels in terms of the clinical characteristics (CRP, WBCs, PLTs) within 24 h after NEC onset in preterm infants (*p* > 0.05). Unhealed NEC included death, abandonment of treatment, and voluntary discharge. In the prognosis of NEC, the results show that there were 2 (18.18%), 3 (23.08%), and 14 (26.42%) unhealed cases in the HHM, LHM, and NHM groups, respectively (*p* = 0.922). Although this prognosis showed that there were no differences among these three groups, disease healing rose with increased HM intake.

## 4. Discussion

NEC and the risk factors associated with it have been an area of interest in neonatology for decades. Necrotizing enterocolitis (NEC) is a multifactor disease affecting preterm infants, including hypercoagulability, shock, cyanosis congenital heart disease, perinatal hypoxia, septicemia, and improper feeding. Current studies suggest that HM is a protective factor against NEC. Despite the proven benefits of exclusive and greater proportions of HM of the total enteral intake for reducing the risk of NEC, many mothers may be unable to provide sufficient HM in the first days after delivery. As a result, mixed feeding is a common practice for preterm infants when HM is insufficient. The main findings of our study support the hypothesis that different volumes of HM intake of the total enteral intake were associated with the occurrence, development, and prognosis of NEC, and HHM case results were most pronounced in preterm infants.

HM and its related substances have been popular in neonatology research. HM can promote intestinal immune function and protect against related complications associated with preterm birth, such as NEC and sepsis [8,26]. In recent years, existing studies have mainly focused on the associations between feeding methods and NEC. Cortez et al. [8] concluded that HM feeding progressed more rapidly and was associated with fewer NEC diagnoses than preterm formula feeding. Cerasani et al. [21] showed that HM-fed NEC cases had a better prognosis than formula feeding for preterm infants. The above studies have indicated that HM plays a protective role against NEC. However, the achievement of exclusive HM feeding can be interfered with by multiple factors, and the association between HM proportion of the total enteral intake and the prognosis of NEC remains unclear.

In the present study, by evaluating risk factors for NEC, a novel influencing factor for the development of NEC was identified: HM proportion. Previous studies and our findings show that the occurrence of NEC in preterm infants might be associated with different HM proportions [13,27]. In a study of 223 very low-birth-weight (VLBW) infants in the U.S., Sisk et al. [19] reported that incidence of NEC was significantly negatively associated with HM proportion of the total enteral feeding in the first 14 days of life (OR = 0.62; 95% CI, 0.51–0.77). Similarly, Chowning et al. [28] reported in 797 VLBW neonates in the U.S. significantly lower incidence and severity of NEC in infants fed high HM proportions than those fed low HM proportions (*p* < 0.05). However, we found that most of the available studies have focused on the association of the occurrence of NEC at a 50% HM proportion and less on the associations after the diagnosis of NEC. The possible reasons for this could be as follows: (i) a large source of variation in studies derived from HM exposure, with most studies choosing to use 50% as a classification focused on early feeding during neonatal admission, and (ii) study population, with some studies choosing to study only VLBW cases, which are a more vulnerable part of our population of interest and may limit applicability. Therefore, our study further explored the associations between the occurrence, development, and prognosis of NEC in preterm infants with a predominantly HM proportion (>70%) of the total enteral intake before the diagnosis of NEC.

Our findings are consistent with recent studies, where the outcome of NEC may be associated with HM intake. The outcome included clinical characteristics, disease severity, and prognosis. In clinical characteristics, preterm infants with NEC had a higher CRP level at 24 h after NEC onset in lower HM proportions and higher HM proportions of the enteral feeds during hospitalization, suggesting a more extended pro-inflammatory response in Gram-negative NEC-associated infections. We also noted lower WBCs, Ns, and platelets in preterm neonates with LHM intake following NEC onset, suggesting the involvement of these cell lines in NEC pathogenesis and outcomes. However, the potential mechanism by which low HM increases the risk of NEC still needs to be further explored.

Our study and previous studies show significant differences in weight gain rate during hospitalization with different HM proportions of the total enteral feeding [19]. These findings suggest that high HM intake is associated with a faster weight growth rate. Our study supports that the timing of the onset and diagnosis of NEC correlate with HM proportion, with the majority of NEC rates occurring in the early stage (i.e., before 14 days) [29,30]. In preterm neonate cohorts, up to 40% occur within this time frame. These observations are consistent with our study, where we included 53 (69%) of the 77 preterm infants in early life. Moreover, we found that later onset and diagnosis were associated with HHM intake, which may be directly related to the protective effects of HM on the gut and against the disease [7]. The timing of NEC onset and diagnosis have important implications for clinical identification and disease progression and may point to different severities of NEC. Our results are in line with those of Cristofalo et al. [31], where HM proportion was associated with the severity of NEC. In our study, the NHM group had higher rates of surgical treatment, severe NEC, and unhealed NEC, suggesting that the NHM group was more severe in terms of the severity of NEC. Current studies suggest that the TLR-4 signaling pathway is an important link in the development of NEC through excessive activation, causing intestinal damage and systemic inflammatory responses [17]. HM is an effective regulator of TLR-4 expression, helping to reduce the symptoms and severity of NEC.

For NEC in preterm infants, hospitalization costs are a heavy burden on families and society. The research of Lewis et al. [32], who observed that different volumes of HM intake were significantly associated with hospitalization costs, is in line with our results, with the HHM group having fewer hospitalization costs than the LHM group. However, the data also showed that the hospitalization costs of the HHM group were higher than the NHM group. The reason for this may be related to the shorter length of stay in the NHM group due to death or abandonment of treatment, etc.

To our knowledge, our study is the first to demonstrate that, if the proportion of HM of the total enteral intake in the total enteral feeding before NEC onset reached more than 70%, it may be associated with reduced occurrence and development of NEC in preterm infants. Even when exclusive HM is not feasible, striving to achieve more than 70% HM intake could have a significant protective effect on the occurrence and development of NEC. However, we acknowledge that there are limitations of this study. One is that, although the information in the database was prospectively collected, it did require retrospective review to identify cases and controls. Another limitation is that we limited our study population to preterm infants in a single NICU. Despite these limitations, there are several important implications of our study. For clinicians, the importance of enhancing HM during preterm infants’ hospitalization should be emphasized. HM intake of more than 70% of the total amount of enteral feeding could be a suggestion to reduce the occurrence and development of NEC. For mothers of preterm infants, the findings of this study can provide evidence to promote human milk expression and/or direct breastfeeding intentions to those who are not willing to breastfeed. Our findings also provide guidance and reference for future research on the role and mechanism of HM in the development of NEC in preterm neonates.

## 5. Conclusions

Our current study contributes valuable evidence supporting the association between HM intake and the prognosis of NEC in preterm infants. A greater proportion of HM of the total enteral feeding appeared to play a protective role in reducing the occurrence of NEC. Moreover, preterm infants who received more than 70% HM in their total enteral feeding demonstrated milder disease severity and a better prognosis. Unfortunately, due to the retrospective case–control design, we still lack a definite explanation as to the potential mechanisms of the HM proportion and the development of NEC. Further studies (e.g., well-designed prospective RCTs) are still needed to elucidate the risk factors and pathogenesis of NEC.

## Figures and Tables

**Table 1:** General characteristics of NEC cases and controls.

Characteristics	NEC (n = 77)	Control (n = 154)	Test	*p* Value ^#^
	n (%) or Mean (SD)	n (%) or Mean (SD)		
Matched parameters				
Male, sex	47(61.04%)	94(61.04%)	χ^2^ = 0.036	0.849
GA, weeks	31.68 ± 2.60	31.77 ± 2.55	t = −0.256	0.798
BW, grams	1660.65 ± 481.36	1654.74 ± 483.02	t = 0.088	0.930
Maternal factors				
Natural birth delivery	16(20.78%)	40(25.97%)	χ^2^ = 0.754	0.385
Prenatal GC therapy	54(70.13%)	98(63.64%)	χ^2^ = 0.962	0.327
GDM	15(19.48%)	25(16.23%)	χ^2^ = 0.378	0.539
PHI	18(23.38%)	33(21.43%)	χ^2^ = 0.113	0.736
Neonatal factors				
Exclusive breastfeeding	24(31.17%)	62(40.26%)	χ^2^ = 1.005	0.316
First feeding ^a^, days	2.38 ± 1.79	2.69 ± 1.45	t = −1.419	0.157
ARDS	28(36.35%)	56(36.36%)	χ^2^ = 0.000	1.000
PDA	23(29.87%)	63(40.91%)	χ^2^ = 2.677	0.102
Neonatal asphyxia	18(23.38%)	58(37.66%)	χ^2^ = 4.746	0.029 *

GA: gestational age; BW: birth weight; GC: glucocorticoid; GDM: gestational diabetes mellitus; PHI: pregnancy-induced hypertension syndrome; ARDS: acute respiratory distress syndrome; PDA: patent ductus arteriosus. ^#^ Welch’s t or chi-squared across all groups. * *p* < 0.05. ^a^ Time of first feeding after birth (days). Results are presented as frequencies (n) and percentages (%) for categorical variables and means and standard deviations (SDs) for normally distributed numeric data.

**Table 2:** The associations of different human milk proportions and the occurrence of NEC (primary outcome).

Human Milk Intake	NEC (n = 77)	Control (n = 154)	OR (95% CI)	Adjusted OR (95% CI)
NHM (0%), n (%)	53(68.83%)	102(66.23%)	1.00	
LHM (<70%), n (%)	13(16.88%) ^a^	19(12.34%)	1.257(0.565–2.799)	1.045(0.452–2.417)
HHM (≥70%), n (%)	11(14.29%) ^a^	33(21.43%)	0.599(0.261–1.377)	0.573(0.246–1.336)
*p* value ^#^			0.317	0.386

Adjusted included gestational age, birth weight, and gender. HHM: high human milk; LHM: low human milk; NHM: no human milk. ^a^ Compared to the NHM group. ^#^ Conditional logistic regression analysis. Results are presented as frequencies (n) and percentages (%) for categorical variables.

**Table 3:** The associations of different human milk proportions with the outcome of NEC (primary outcome).

Parameter		Grouped by Proportion of Human Milk to Total Enteral Feeding			*p* Value ^#^
	All subjects	≥70%	<70%	0%	
	n = 77	n = 11	n = 13	n = 53	
CRP (mg/mL)		9.10 ± 12.58	16.09 ± 26.47	28.99 ± 53.37	0.312
WBCs (×10^9^/L)		12.76 ± 6.28	16.24 ± 10.67	14.44 ± 9.56	0.634
N (%)		41.25 ± 19.58	50.27 ± 19.22	48.31 ± 21.43	0.545
PLTs (×10^9^/L)		281.60 ± 139.37	270.38 ± 145.31	263.78 ± 164.68	0.960
Weight gain (g/kg^−1^/day^−1^) ^a^		14.99 ± 9.41	9.35 ± 4.14	8.99 ± 6.88	0.035 *
Onset day (d)		21.9 ± 12.9	17.9 ± 11.2	11.7 ± 7.3	0.024 *
Diagnosis day (d)		25.9 ± 11.7	19.8 ± 11.3	13.9 ± 7.9	0.003 *
Hospital day (d)		31.1 ± 12.5	36.3 ± 16.5	29.2 ± 19.8	0.275
Hospitalization cost (CNY)		77,802.46 ± 29,454.21	95,554.65 ± 47,002.48	64,773.89 ± 49,409.47	0.030 *
Surgical treatment, n (%)		3(27.27%)	6(46.15%)	17(32.08%)	0.620
Duration of antibiotic use (d)		10.5 ± 4.8	10.5 ± 7.5	9.8 ± 6.8	0.902
Severity of NEC, n (%) ^b^					
Mild ^c^		9(81.82%)	9 (69.23%)	21(39.62%)	0.013 *
Severe ^d^		2(18.18%)	4 (30.77%)	32(60.38%)	
Prognosis of NEC, n (%)					
Improvement ^e^		9(81.82%)	10 (76.92%)	39(73.58%)	0.922
Not healed ^f^		2(18.18%)	3 (23.08%)	14(26.42%)	

^#^ Chi-squared, Fisher’s exact, or ANOVA tests across all groups. * *p* < 0.05. C-reaction (CRP); white blood cells (WBCs); neutrophil (N); platelets (PLTs); day (d); Chinese Yuan (CNY). ^a^ Weight gain = [1000 × ln(Wt_2_ − Wt_1_)]/T (Wt_1_: admission weight; Wt_2_: weight at the end of hospitalization; weight gain was the growth rate during hospitalization). ^b^ Severity of NEC was categorized according to Bell’s classification standard [24]. ^c^ NEC I and NEC II A were classified as “mild” stages. ^d^ NEC II B and NEC III were “severe” stages. ^e^ Improvement included improvement or cure of disease. ^f^ Not healed included unhealed or abandonment of treatment, etc. Results are presented as frequencies (n) and percentages (%) for categorical variables and means and standard deviations (SDs) for normally distributed numeric data.

## Data Availability

The datasets generated during and/or analyzed during the current study are available from the corresponding author on reasonable request.
